# Supplementary material for: Optical Response Characteristics of Single-Walled Carbon Nanotube Chirality Exposed to Oxidants with Different Oxidizing Power
Source: Molecules. 2021 Feb 19;26(4):1091. doi: 10.3390/molecules26041091 (PMC7922499; doi:10.3390/molecules26041091)
Supplement: Supplementary file 1 [file molecules-26-01091-s001.pdf]

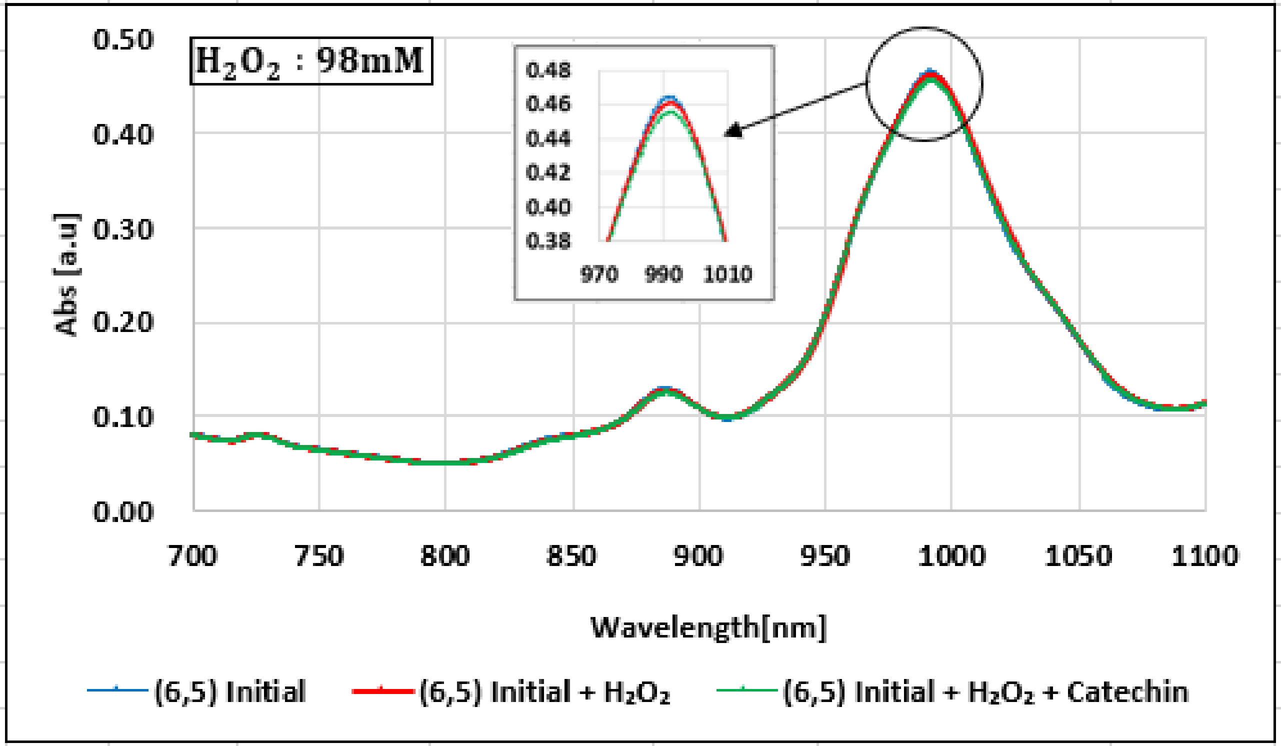

Sample	Peak Abs: Chirality (6,5)				
	Initial State	H_2O_2 Addition	Difference	Catechin Addition	Difference
H_2O_2 (98mM)	0.4642	0.4609	-0.72%	0.4550	-1.27%
	Peak Wavelength (nm)				
	992.0	992.0	0.0	993.0	1.0

Figure S1. Absorption spectra of dsDNA– (6,5)-Enriched SWNT Complex by H_2O_2

The absorption spectra measured with 98mM of H_2O_2concentration are shown.
The data are presented as the average of three independent experiments.

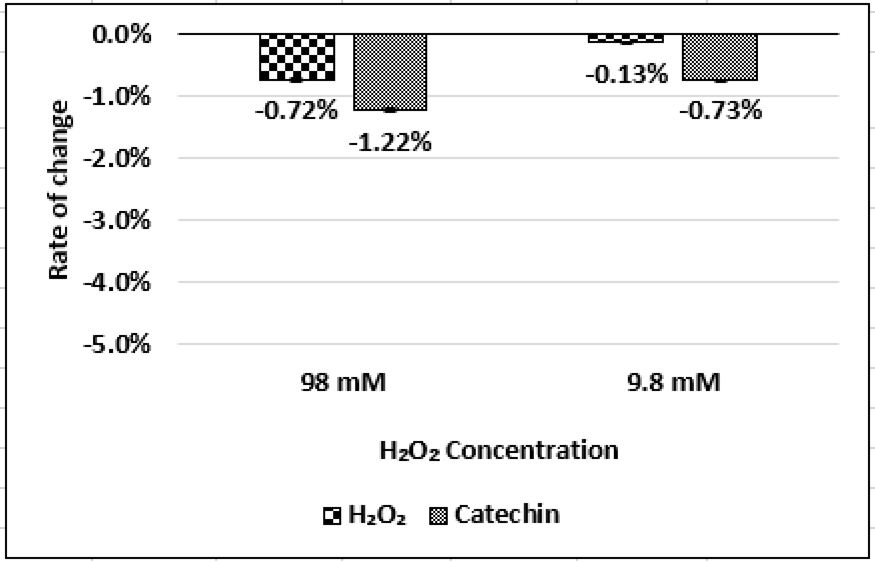

Concentration	dsDNA- (6,5) enriched SWNT Absorbance				
(H2O2)	Initial State	H_2O_2 Addition	Change rate from Initial state	Catechin addition	Change rate from H_2O_2 Addition
98 mM	0.4642 ± 0.0004	0.4609 ± 0.0002	-0.72 ± 0.03%	0.4552 ± 0.0001	-1.22 ± 0.03%
9.8 mM	0.4657 ± 0.0001	0.4650 ± 0.0001	-0.13 ± 0.01%	0.4616 ± 0.0001	-0.73 ± 0.00%

Concentration	dsDNA- (6,5) enriched SWNT Peak Wavelength (nm)				
(H2O2)	Initial State	H_2O_2 Addition	Shift from Initial state	Catechin addition	Shift from Initial state
98 mM	992.0± 0.000	992.0± 0.000	0.0 ± 0.000	992.3 ± 0.471	0.3 ± 0.385
9.8 mM	992.0± 0.000	992.0± 0.000	0.0 ± 0.000	992.0± 0.000	0.0 ± 0.000

Figure S2. Absorbance rate of change and wavelength peak shift of dsDNA- (6,5) enriched SWNT Complex by H_2O_2

At the final concentrations of 98 mM and 9.8 mM, no significant spectral change was observed when H_2O_2 was added.
There was no significant difference in the rate of change when catechin was added thereafter.
No significant change was observed in the wavelength peak shift.
The data are presented as the average of three independent experiments.

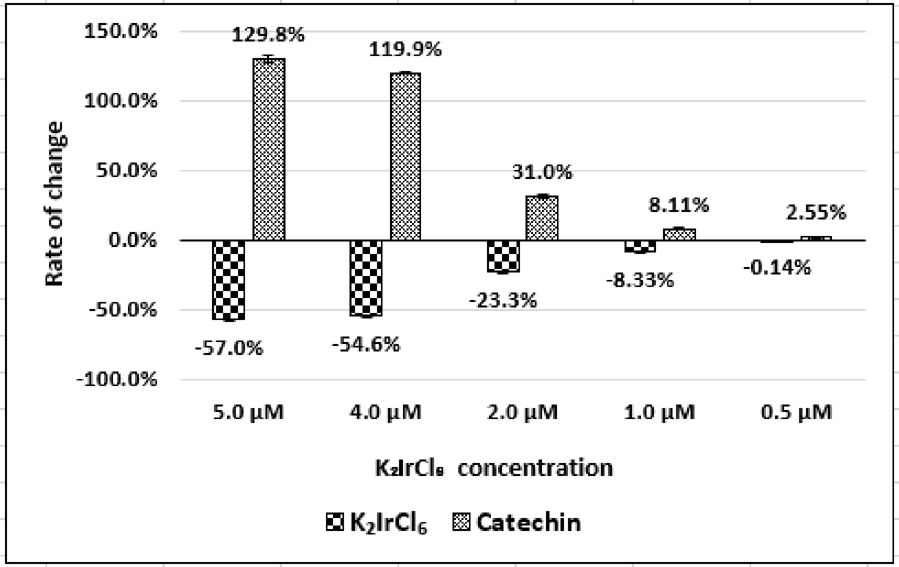

Concentration	dsDNA- (6,5) enriched SWNT Absorbance				
(K_2IrCl_6)	Initial State	K_2IrCl_6 Addition	Change rate from Initial state	Catechin addition	Change rate from K_2IrCl_6 Addition
5.0 μM	0.4281 ± 0.0001	0.1842 ± 0.0024	-57.0 ± 0.45%	0.4233 ± 0.0000	129.8 ± 2.47%
4.0 μM	0.4289 ± 0.0003	0.1932 ± 0.0009	-54.6 ± 0.14%	0.4249 ± 0.0003	119.9 ± 0.66%
2.0 μM	0.4268 ± 0.0002	0.3272 ± 0.0030	-23.3 ± 0.55%	0.4286 ± 0.0000	31.0± 0.97%
1.0 μM	0.4268 ± 0.0007	0.3912 ± 0.0009	-8.33 ± 0.04%	0.4229 ± 0.0000	8.11 ± 0.19%
0.5 μM	0.4265 ± 0.0010	0.4259 ± 0.0007	-0.14 ± 0.05%	0.4368 ± 0.0002	2.55± 0.10%

Concentration	dsDNA- (6,5) enriched SWNT Peak Wavelength (nm)				
(K_2IrCl_6)	Initial State	K_2IrCl_6 Addition	Shift from Initial state	Catechin addition	Shift from Initial state
5.0 μM	994.0 ± 0.000	999.7 ± 0.471	5.7 ± 0.385	995.0 ± 0.000	1.0 ± 0.000
4.0 μM	994.3 ± 0.471	1000 ± 0.000	5.7 ± 0.385	995.0 ± 0.000	0.7 ± 0.385
2.0 μM	994.0 ± 0.000	994.0 ± 0.000	0.0 ± 0.000	995.0 ± 0.000	1.0 ± 0.000
1.0 μM	994.3 ± 0.471	994.0 ± 0.000	-0.3 ± 0.385	995.0 ± 0.000	0.7 ± 0.385
0.5 μM	994.7 ± 0.471	994.3 ± 0.471	-0.3 ± 0.000	995.0 ± 0.000	0.3 ± 0.385

Figure S3. Absorbance rate of change and wavelength peak shift of dsDNA- (6,5) enriched SWNT Complex by $K_2I_rCl_6$

The decrease in absorbance proportional to the concentration was detected in the concentration range of 0.5 to 5.0 μM. The rate of change at 5 μM was -57.0%. In the same range, recovery of absorbance by addition of catechin was observed. The rate of change at 5 μM was 129.8%. At the concentration of $K_2I_rCl_6$ of 5.0 μM, the peak wavelength was shifted 5.7 nm to the short wavelength side. It recovered to near the initial state with the addition of catechin. The data are presented as the average of three independent experiments.

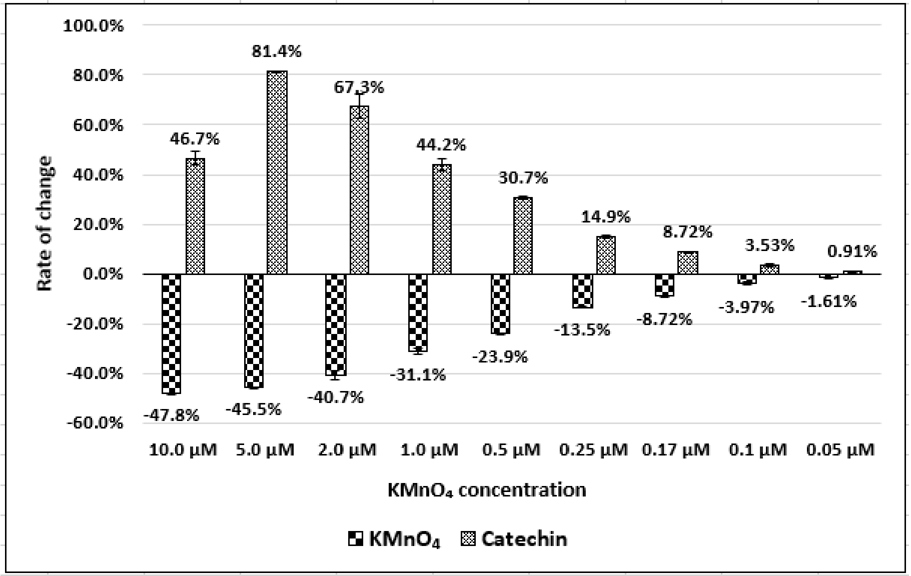

Concentration	dsDNA- (6,5) enriched SWNT Absorbance				
($KMnO_4$)	Initial State	$KMnO_4$ Addition	Change rate from Initial state	Catechin addition	Change rate from $KMnO_4$
10.0 µM	0.4790 ± 0.0013	0.2500 ± 0.0007	-47.8 ± 0.00%	0.3667 ± 0.0086	46.7 ± 2.47%
5.0 µM	0.4640 ± 0.0008	0.2529 ± 0.0004	-45.5 ± 0.00%	0.4587 ± 0.0013	81.4 ± 0.15%
2.0 µM	0.4629 ± 0.0011	0.2744 ± 0.0101	-40.7 ± 1.67%	0.4590 ± 0.0005	67.3 ± 4.89%
1.0 µM	0.4655 ± 0.0029	0.3206 ± 0.0089	-31.1 ± 1.22%	0.4622 ± 0.0032	44.2± 2.46%
0.5 µM	0.4657 ± 0.0043	0.3543 ± 0.0043	-23.9 ± 0.18%	0.4630 ± 0.0037	30.7 ± 0.43%
0.25 µM	0.4693 ± 0.0038	0.4060 ± 0.0051	-13.5 ± 0.31%	0.4666 ± 0.0040	14.9± 0.38%
0.17 µM	0.4661 ± 0.0052	0.4255 ± 0.0041	-8.72 ± 0.11%	0.4626 ± 0.0051	8.72± 0.11%
0.10 µM	0.4675 ± 0.0033	0.4489 ± 0.0006	-3.97 ± 0.43%	0.4648 ± 0.0035	3.53± 0.51%
0.05 µM	0.4670 ± 0.0031	0.4595 ± 0.0031	-1.61 ± 0.01%	0.4637 ± 0.0030	0.91± 0.03%

Concentration	dsDNA- (6,5) enriched SWNT Peak Wavelength (nm)				
($KMnO_4$)	Initial State	$KMnO_4$ Addition	Shift from Initial state	Catechin addition	Shift from Initial state
10.0 µM	992.0 ± 0.000	975.3 ± 0.471	-16.7 ± 0.385	987.7 ± 0.471	-4.3 ± 0.385
5.0 µM	992.0 ± 0.000	975.3 ± 0.471	-16.7 ± 0.385	992.0 ± 0.000	0.0 ± 0.000
2.0 µM	992.0 ± 0.000	978.3 ± 1.247	-13.7 ± 1.018	992.0 ± 0.000	0.0 ± 0.000
1.0 µM	992.0 ± 0.000	985.7 ± 1.886	-6.3 ± 1.540	992.0 ± 0.000	0.0 ± 0.000
0.5 µM	992.0 ± 0.000	988.0 ± 0.816	-4.0 ± 0.667	992.0 ± 0.000	0.0 ± 0.000
0.25 µM	992.0 ± 0.000	990.0 ± 0.000	-2.0 ± 0.000	992.0 ± 0.000	0.0 ± 0.000
0.17 µM	992.0 ± 0.000	991.0 ± 0.000	-1.0 ± 0.000	992.0 ± 0.000	0.0 ± 0.000
0.10 µM	992.0 ± 0.000	991.3 ± 0.471	-0.7 ± 0.385	992.0 ± 0.000	0.0 ± 0.000
0.05 µM	992.0 ± 0.000	992.0 ± 0.000	0.0 ± 0.000	992.0 ± 0.000	0.0 ± 0.000

Figure S4. Absorbance rate of change and wavelength peak shift of dsDNA- (6,5) enriched SWNT Complex by KM_nO_4

The decrease in absorbance proportional to the concentration was detected in the concentration range of 0.05 to 10.0 µM. The rate of change at 10 µM was -47.8%. In the same range, recovery of absorbance by addition of catechin was observed. The rate of change at 10 µM was 46.7%.
At the concentration of KM_nO_4 of 5.0 µM, the peak wavelength was shifted 16.7 nm to the short wavelength side. By adding catechin, it recovered from the initial state to the position of 4.3 nm on the short wavelength side.
The data are presented as the average of three independent experiments.

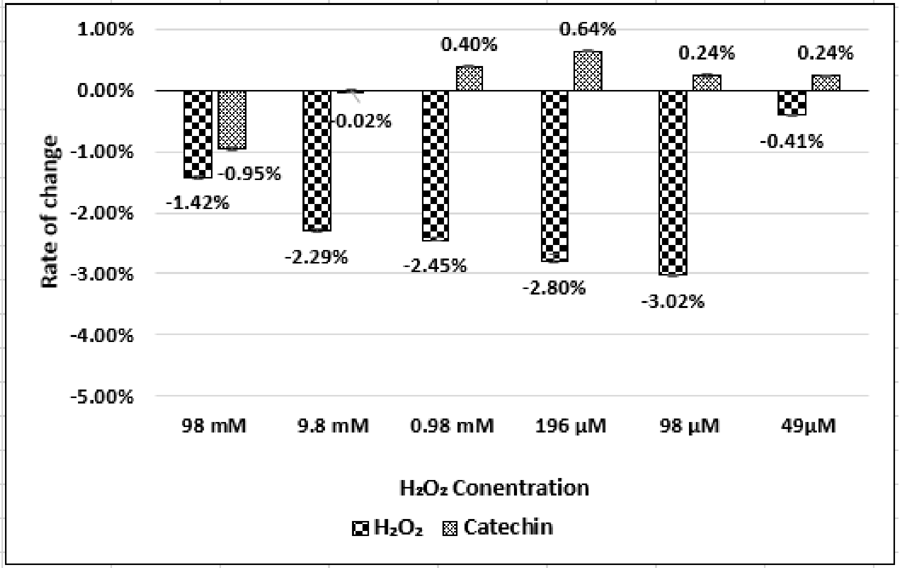

Concentration	dsDNA- HiPco (6,5) SWNT Absorbance				
(H_2O_2)	Initial State	H_2O_2 Addition	Change rate from Initial state	Catechin addition	Change rate from H_2O_2 Addition
98 mL	0.2939 ± 0.0002	0.2897 ± 0.0001	-1.42± 0.04%	0.2869 ± 0.0003	-0.95± 0.03%
9.8 mL	0.3205 ± 0.0002	0.3131 ± 0.0001	-2.29 ± 0.03%	0.3131 ± 0.0002	0.02 ± 0.03%
0.98 mL	0.3212 ± 0.0005	0.3134 ± 0.0001	-2.45 ± 0.05%	0.3146 ± 0.0001	0.40 ± 0.00%
196 µL	0.3230± 0.0005	0.3139 ± 0.0001	-2.80 ± 0.11%	0.3159 ± 0.0000	0.64± 0.01%
98 µL	0.3230 ± 0.0001	0.3133 ± 0.0001	-3.02 ± 0.01%	0.3140 ± 0.0002	0.24 ± 0.02%
49 µL	0.3159 ± 0.0000	0.3146 ± 0.0001	-0.41 ± 0.01%	0.3154 ± 0.0000	0.24± 0.01%

Concentration	dsDNA- HiPco (6,5) SWNT Peak Wavelength (nm)				
(H_2O_2)	Initial State	H_2O_2 Addition	Shift from Initial state	Catechin addition	Shift from Initial state
98 mL	993.0 ± 0.000	993.0 ± 0.000	0.0 ± 0.000	963.0 ± 0.000	0.0± 0.000
9.8 mL	991.3 ± 0.471	991.2 ± 0.236	-0.2 ± 0.192	991.8 ± 0.000	0.5 ± 0.000
0.98 mL	991.5 ± 0.000	991.2 ± 0.236	-0.3 ± 0.192	992.0 ± 0.000	0.7 ± 0.192
196 µL	991.0 ± 0.000	991.2 ± 0.236	0.3 ± 0.192	992.0 ± 0.000	1.0 ± 0.000
98 µL	991.5 ± 0.000	991.5 ± 0.000	0.0 ± 0.000	992.0 ± 0.000	0.5 ± 0.000
49 µL	991.5 ± 0.000	991.5 ± 0.000	0.0 ± 0.000	992.0 ± 0.000	0.5 ± 0.000

Figure S5. Absorbance rate of change and wavelength peak shift of dsDNA- HiPco (6,5) SWNT Complex by H_2O_2

The rate of change in absorbance decreased by up to 3.0% when the H_2O_2 concentration was 98 µM, but no significant difference was observed in the range of 49 µM to 98 mM. There was no significant difference in the rate of change when catechin was added thereafter.
No significant change was observed in the wavelength peak shift.
The data are presented as the average of three independent experiments.

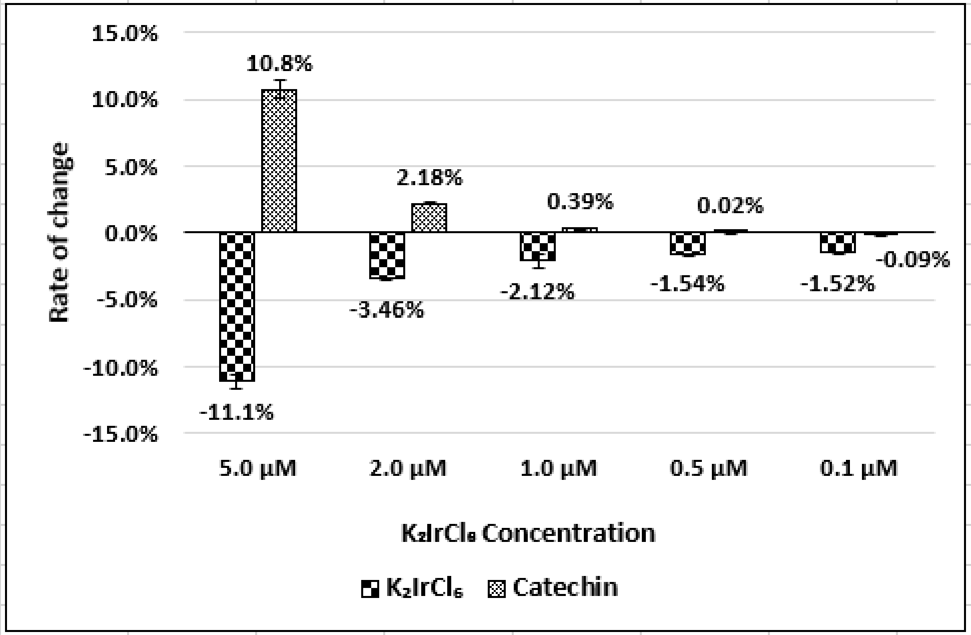

Concentration	dsDNA- HiPco (6,5) SWNT Absorbance				
(K_2IrCl_6)	Initial State	K_2IrCl_6 Addition	Change rate from Initial state	Catechin addition	Change rate from H_2O_2 Addition
5.0 µM	0.4050 ± 0.0000	0.3601 ± 0.0028	-11.1 ± 0.55%	0.3989 ± 0.0000	10.8 ± 0.69%
2.0 µM	0.4057± 0.0002	0.3916 ± 0.0005	-3.46 ± 0.06%	0.4002 ± 0.0000	2.18 ± 0.10%
1.0 µM	0.4058 ± 0.0024	0.3972 ± 0.0001	-2.12 ± 0.46%	0.3988 ± 0.0001	0.39 ± 0.00%
0.5 µM	0.4058 ± 0.0001	0.3996 ± 0.0001	-1.54 ± 0.00%	0.3997 ± 0.0000	0.02 ± 0.00%
0.1 µM	0.4045 ± 0.0003	0.3984 ± 0.0000	-1.52 ± 0.06%	0.3980 ± 0.0000	-0.09 ± 0.00%

Concentration	dsDNA- HiPco (6,5) SWNT Peak Wavelength (nm)				
(K_2IrCl_6)	Initial State	K_2IrCl_6 Addition	Shift from Initial state	Catechin addition	Shift from Initial state
5.0 µM	993.0 ± 0.000	993.0 ± 0.000	0.0 ± 0.000	993.7 ± 0.236	0.7 ± 0.192
2.0 µM	993.0 ± 0.000	992.5 ± 0.000	-0.5 ± 0.000	993.5 ± 0.000	0.5 ± 0.000
1.0 µM	992.5 ± 0.000	992.5 ± 0.000	0.0 ± 0.000	994.0 ± 0.000	1.5 ± 0.000
0.5 µM	993.0 ± 0.000	993.0 ± 0.000	0.0 ± 0.000	994.0 ± 0.000	1.0 ± 0.000
0.1 µM	993.5 ± 0.000	993.5 ± 0.000	0.0 ± 0.000	994.0 ± 0.000	0.5 ± 0.000

Figure S6. Absorbance rate of change and wavelength peak shift of dsDNA- HiPco (6,5) SWNT Complex by $K_2I_rCl_6$

The decrease in absorbance proportional to the concentration was detected in the concentration range of 0.1 to 5.0 µM. The rate of change at 5.0µM was -11.1%. In the same range, recovery of absorbance by addition of catechin was observed. The rate of change at 5.0µM was 10.8%.
No significant change was observed in the wavelength peak shift.
The data are presented as the average of three independent experiments.

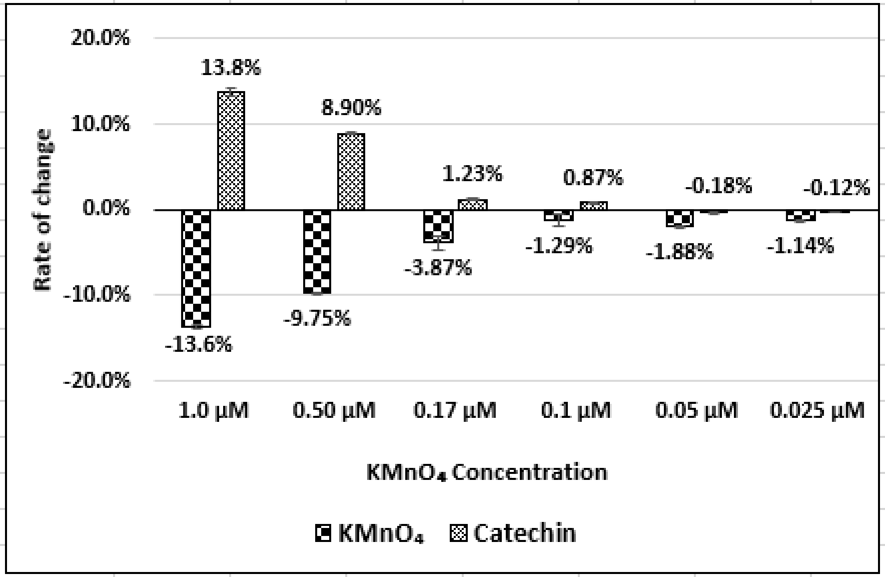

Concentration	dsDNA- HiPco (6,5) SWNT Absorbance				
($KMnO_4$)	Initial State	$KMnO_4$ Addition	Change rate from Initial state	Catechin addition	Change rate from $KMnO_4$ Addition
1.0 µM	0.2880 ± 0.0001	0.2488 ± 0.0011	-13.6 ± 0.30%	0.2830 ± 0.0000	13.8 ± 0.42%
0.50 µM	0.2820 ± 0.0001	0.2545 ± 0.0003	-9.75 ± 0.06%	0.2772 ± 0.0000	8.90 ± 0.10%
0.17 µM	0.2835± 0.0032	0.2725 ± 0.0000	-3.87 ± 0.88%	0.2759 ± 0.0000	1.23 ± 0.01%
0.10 µM	0.2846 ± 0.0024	0.2809 ± 0.0000	-1.29 ± 0.66%	0.2833 ± 0.0000	0.87 ± 0.01%
0.05 µM	0.2969 ± 0.0000	0.2913 ± 0.0000	-1.88 ± 0.00%	0.2908 ± 0.0000	-0.18 ± 0.00%
0.025 µM	0.2898 ± 0.0000	0.2865 ± 0.0000	-1.14 ± 0.00%	0.2862 ± 0.0000	-0.12 ± 0.01%

Concentration	dsDNA- HiPco (6,5) SWNT Peak Wavelength (nm)				
($KMnO_4$)	Initial State	$KMnO_4$ Addition	Shift from Initial state	Catechin addition	Shift from Initial state
1.0 µM	993.0 ± 0.000	995.7 ± 0.236	2.7 ± 0.192	993.5 ± 0.000	0.5 ± 0.000
0.50 µM	992.0 ± 0.000	992.0 ± 0.000	0.0 ± 0.000	992.5 ± 0.000	0.5 ± 0.000
0.17 µM	992.0 ± 0.000	992.0 ± 0.000	0.0 ± 0.000	992.5 ± 0.000	0.5 ± 0.000
0.10 µM	993.0 ± 0.000	992.5 ± 0.000	-0.5 ± 0.000	993.8 ± 0.236	0.8 ± 0.192
0.05 µM	992.5 ± 0.000	992.5 ± 0.000	0.0 ± 0.000	992.5 ± 0.000	0.0 ± 0.000
0.025 µM	992.5 ± 0.000	992.5 ± 0.000	0.0 ± 0.000	993.0 ± 0.000	0.5 ± 0.000

Figure S7. Absorbance rate of change and wavelength peak shift of dsDNA- HiPco (6,5) SWNT Complex by KM_nO_4

The decrease in absorbance proportional to the concentration was detected in the concentration range of 0.025 to 1.0 µM. The rate of change at 1.0µM was -13.6%. In the same range, recovery of absorbance by addition of catechin was observed. The rate of change at 1.0µM was 13.8%. At the concentration of KM_nO_4 of 1.0µM, the peak wavelength was shifted 2.7 nm to the long wavelength side. By adding catechin, it recovered to near the initial state with the addition of catechin.
The data are presented as the average of three independent experiments.

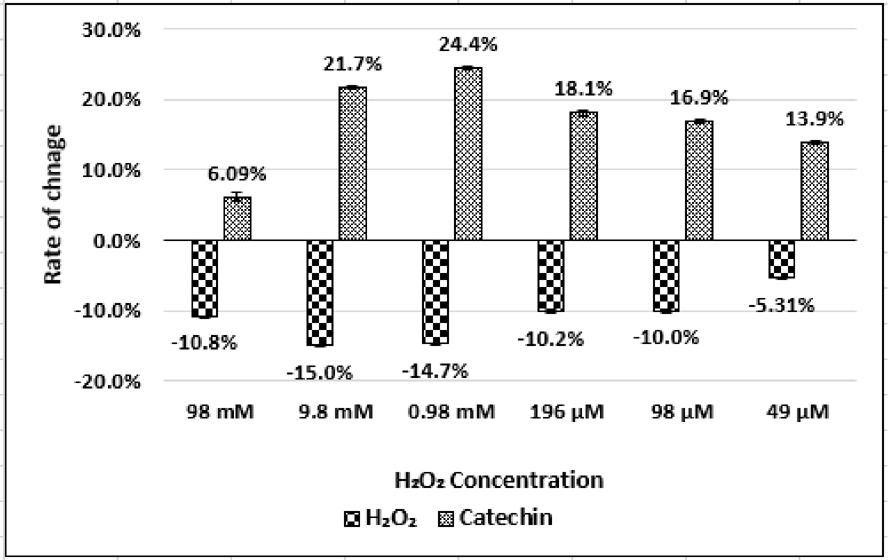

Concentration (H_2O_2)	dsDNA- HiPco (8,7) SWNT Absorbance				
	Initial State	H_2O_2 Addition	Change rate from Initial state	Catechin addition	Change rate from H_2O_2 Addition
98 mL	0.3353 ± 0.0003	0.2991 ± 0.0001	-10.8 ± 0.04%	0.3173 ± 0.0025	6.09± 0.66%
9.8 mL	0.3661 ± 0.0003	0.3112 ± 0.0003	-15.0 ± 0.01%	0.3787 ± 0.0012	21.7± 0.24%
0.98 mL	0.3657 ± 0.0009	0.3119 ± 0.0008	-14.7 ± 0.02%	0.3880 ± 0.0004	24.4 ± 0.17%
196 µL	0.3704± 0.0007	0.3328 ± 0.0016	-10.2 ± 0.21%	0.3929 ± 0.0001	18.1± 0.43%
98 µL	0.3702 ± 0.0002	0.3331 ± 0.0014	-10.0 ± 0.27%	0.3893 ± 0.0003	16.9 ± 0.34%
49 µL	0.3626 ± 0.0008	0.3434 ± 0.0012	-5.31 ± 0.09%	0.3912 ± 0.0001	13.9± 0.30%

Concentration (H_2O_2)	dsDNA- HiPco (8,7) SWNT Peak Wavelength (nm)				
	Initial State	H_2O_2 Addition	Shift from Initial state	Catechin addition	Shift from Initial state
98 mL	1266.5 ± 0.000	1262.0 ± 0.000	-4.5 ± 0.000	1266.2 ± 0.236	-0.3± 0.192
9.8 mL	1266.7 ± 0.236	1262.0 ± 0.000	-4.7 ± 0.192	1269.0 ± 0.000	2.3 ± 0.192
0.98 mL	1266.8 ± 0.236	1262.0 ± 0.000	-4.8 ± 0.192	1270.5 ± 0.000	3.7 ± 0.192
196 µL	1266.7 ± 0.236	1264.0 ± 0.408	-2.7 ± 0.141	1270.8 ± 0.624	4.2 ± 0.317
98 µL	1267.2 ± 0.236	1264.5 ± 0.408	-2.7 ± 0.141	1270.7 ± 0.236	3.5 ± 0.000
49 µL	1267.0 ± 0.408	1265.5 ± 0.408	-1.5 ± 0.000	1270.8 ± 0.236	3.8 ± 0.141

Figure S8. Absorbance rate of change and wavelength peak shift of dsDNA- HiPco (8,7) SWNT Complex by H_2O_2

When the H_2O_2 concentration changed from 49 µM to 9.8 mM in step by step, the rate of change in absorbance increased, but at 98 mM, the rate of change decreased. The rate of change at 0.98mM was -14.7%. %. In the same range, recovery of absorbance by addition of catechin was observed. The rate of change at 0.98mM was 24.4%.

At the concentration of H_2O_2 of 0.98mM, the peak wavelength was shifted 4.8 nm to the short wavelength side. By adding catechin, it recovered from the initial state to the position of 3.7 nm on the long wavelength side.

The data are presented as the average of three independent experiments.

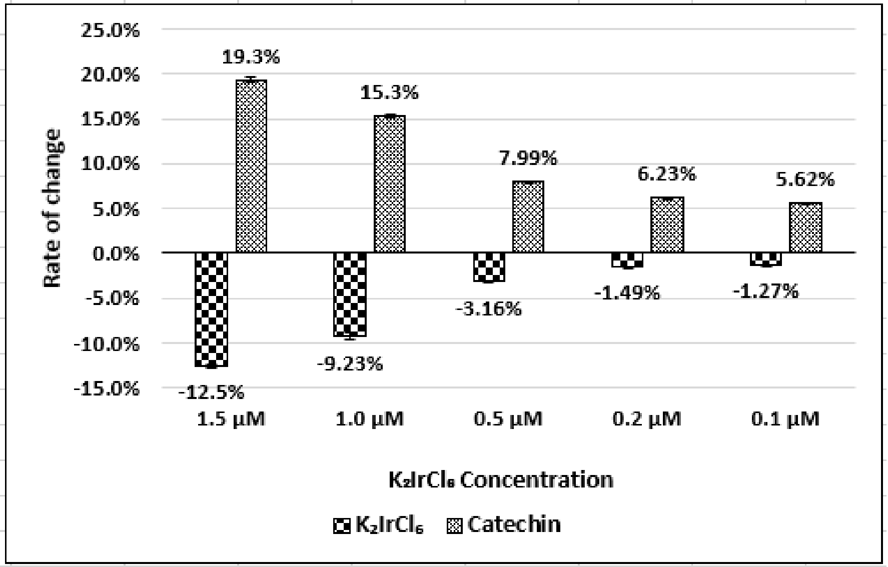

Concentration	dsDNA- HiPco (8,7) SWNT Absorbance				
(K_2IrCl_6)	Initial State	K_2IrCl_6 Addition	Change rate from Initial state	Catechin addition	Change rate from H_2O_2 Addition
1.5 µM	0.4650 ± 0.0005	0.4066 ± 0.0012	-12.5 ± 0.13%	0.4853 ± 0.0000	19.3± 0.28%
1.0 µM	0.4614 ± 0.0034	0.4118 ± 0.0007	-9.23 ± 0.41%	0.4827 ± 0.0000	15.3 ± 0.16%
0.5 µM	0.4627 ± 0.0001	0.4481 ± 0.0004	-3.16 ± 0.06%	0.4839 ± 0.0000	7.99 ± 0.08%
0.2 µM	0.4611± 0.0003	0.4542 ± 0.0002	-1.49 ± 0.01%	0.4825 ± 0.0001	6.23 ± 0.03%
0.1 µM	0.4619 ± 0.0001	0.4561 ± 0.0001	-1.27 ± 0.01%	0.4817 ± 0.0001	5.62 ± 0.02%

Concentration	dsDNA- HiPco (8,7) SWNT Peak Wavelength (nm)				
(K_2IrCl_6)	Initial State	K_2IrCl_6 Addition	Shift from Initial state	Catechin addition	Shift from Initial state
1.5 µM	1267.0 ± 0.000	1261.3 ± 0.236	-5.7 ± 0.192	1271.0 ± 0.000	4.0± 0.000
1.0 µM	1266.5 ± 0.000	1261.3 ± 0.236	-3.7 ± 0.192	1270.5 ± 0.000	4.0± 0.000
0.5 µM	1267.0 ± 0.000	1266.2 ± 0.236	-0.8 ± 0.192	1270.7 ± 0.236	3.7 ± 0.192
0.2 µM	1267.0 ± 0.000	1267.0 ± 0.000	0.0 ± 0.000	1270.7 ± 0.236	3.7 ± 0.192
0.1 µM	1267.0 ± 0.000	1267.3 ± 0.236	0.3 ± 0.192	1271.0 ± 0.000	4.0 ± 0.000

Figure S9. Absorbance rate of change and wavelength peak shift of dsDNA- HiPco (8,7) SWNT Complex by $K_2I_rCl_6$

The decrease in absorbance proportional to the concentration was detected in the concentration range of 0.1 to 1.5 µM. . The rate of change at 1.5µM was -12.5%. In the same range, recovery of absorbance by addition of catechin was observed. The rate of change at 1.5µM was 19.3%. At the concentration of $K_2I_rCl_6$ of 1.5µM, the peak wavelength was shifted 5.7 nm to the short wavelength side. By adding catechin, it recovered from the initial state to the position of 4.0 nm on the long wavelength side.
The data are presented as the average of three independent experiments.

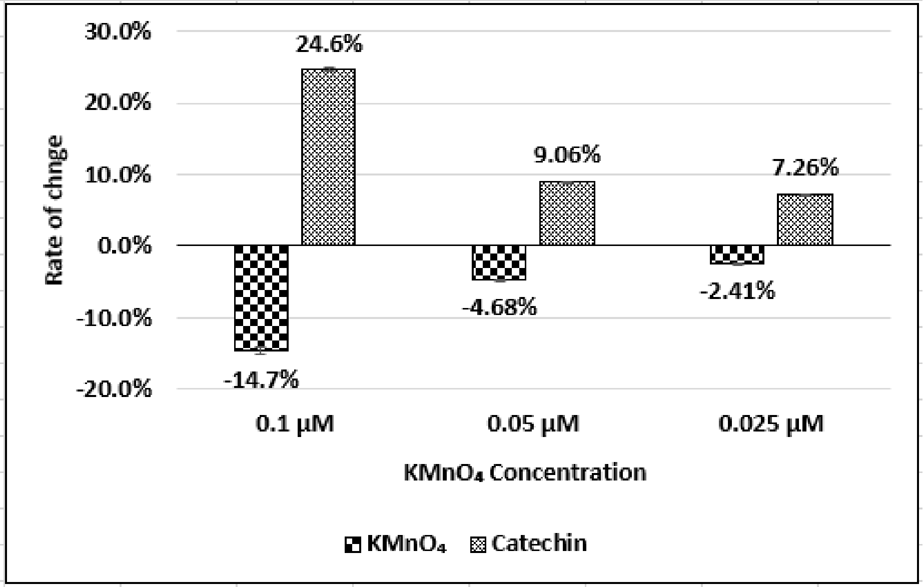

**Concentration**	**dsDNA- HiPco (8,7) SWNT Absorbance**				
**($KMnO_4$)**	**Initial State**	**$KMnO_4$ Addition**	**Change rate from Initial state**	**Catechin addition**	**Change rate from $KMnO_4$ Addition**
**0.10 μM**	0.3220 ± 0.0031	0.2747 ± 0.0007	-14.7 ± 0.50%	0.3424 ± 0.0000	24.6 ± 0.24%
**0.05 μM**	0.3448± 0.0003	0.3287 ± 0.0001	-4.68 ± 0.04%	0.3585 ± 0.0000	9.06 ± 0.03%
**0.025 μM**	0.3369 ± 0.0003	0.3288 ± 0.0001	-2.41 ± 0.05%	0.3527 ± 0.0000	7.26 ± 0.01%

**Concentration**	**dsDNA- HiPco (8,7) SWNT Peak Wavelength (nm)**				
**($KMnO_4$)**	**Initial State**	**$KMnO_4$ Addition**	**Shift from Initial state**	**Catechin addition**	**Shift from Initial state**
**0.10 μM**	1267.3 ± 0.471	1261.2 ± 0.850	-6.2 ± 0.309	1270.5 ± 0.408	3.2 ± 0.052
**0.05 μM**	1266.8 ± 0.471	1266.2 ± 0.624	-0.7 ± 0.124	1270.7 ± 0.624	3.8 ± 0.124
**0.025 μM**	1267.0 ± 0.408	1267.0 ± 0.408	0.0 ± 0.000	1270.7 ± 0.624	3.7 ± 0.176

Figure S10. Absorbance rate of change and wavelength peak shift of dsDNA- HiPco (8,7) SWNT Complex by KM_nO_4

The decrease in absorbance proportional to the concentration was detected in the concentration range of 0.025 to 0.1 μM. The rate of change at 0.1μM was -14.7%. In the same range, recovery of absorbance by addition of catechin was observed. The rate of change at 0.1μM was 24.6%.
At the concentration of KM_nO_4 of 0.1μM, the peak wavelength was shifted 6.2 nm to the short wavelength side. By adding catechin, it recovered from the initial state to the position of 3.2 nm on the long wavelength side.
The data are presented as the average of three independent experiments.

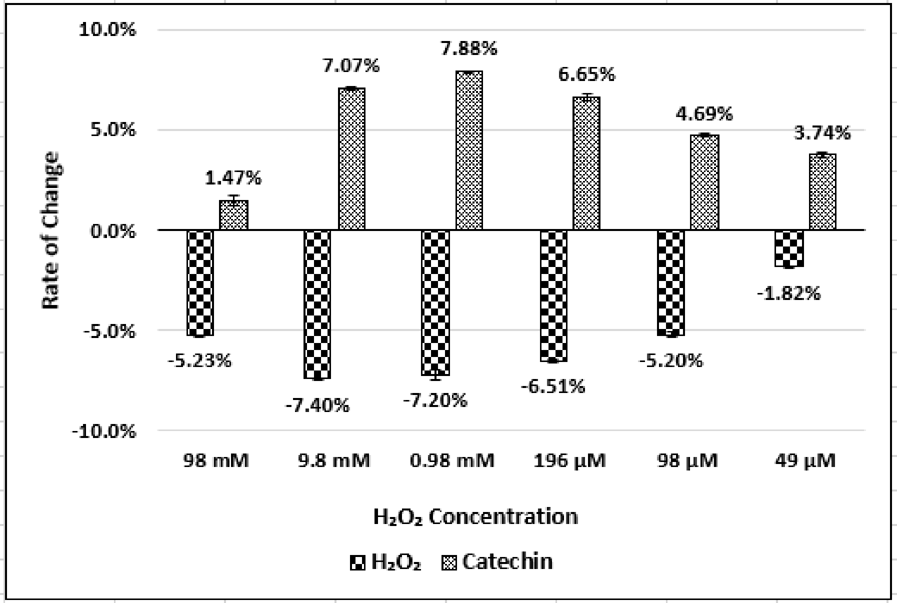

Concentration	dsDNA- HiPco (9,4) SWNT Absorbance				
(H_2O_2)	Initial State	H_2O_2 Addition	Change rate from Initial state	Catechin addition	Change rate from H_2O_2 Addition
98 mL	0.3954 ± 0.0005	0.3747 ± 0.0001	-5.23 ± 0.07%	0.3802 ± 0.0011	1.47 ± 0.23%
9.8 mL	0.4494 ± 0.0002	0.4161 ± 0.0001	-7.40 ± 0.02%	0.4455 ± 0.0005	7.07 ± 0.08%
0.98 mL	0.4491 ± 0.0018	0.4168 ± 0.0004	-7.20 ± 0.23%	0.4496 ± 0.0002	7.88 ± 0.04%
196 μL	0.4537 ± 0.0005	0.4241 ± 0.0009	-6.51 ± 0.07%	0.4523 ± 0.0000	6.65 ± 0.17%
98 μL	0.4534 ± 0.0001	0.4298 ± 0.0007	-5.20 ± 0.11%	0.4500 ± 0.0003	4.69 ± 0.10%
49 μL	0.4438 ± 0.0002	0.4357 ± 0.0005	-1.82 ± 0.06%	0.4520 ± 0.0000	3.74 ± 0.09%

Concentration	dsDNA- HiPco (9,4) SWNT Peak Wavelength (nm)				
(H_2O_2)	Initial State	H_2O_2 Addition	Shift from Initial state	Catechin addition	Shift from Initial state
98 mL	1134.5 ± 0.000	1133.7 ± 0.236	-0.8 ± 0.192	1134.5 ± 0.000	0.0± 0.000
9.8 mL	1133.8 ± 0.236	1133.7 ± 0.236	-0.2 ± 0.000	1133.7 ± 0.236	-0.2± 0.000
0.98 mL	1133.5 ± 0.000	1133.5 ± 0.000	0.0 ± 0.000	1134.0 ± 0.000	0.5± 0.000
196 μL	1133.0 ± 0.000	1134.0 ± 0.000	1.0 ± 0.000	1134.5 ± 0.000	1.5± 0.000
98 μL	1133.5 ± 0.000	1133.5 ± 0.000	0.0 ± 0.000	1134.0 ± 0.000	0.5± 0.000
49 μL	1133.5 ± 0.000	1133.5 ± 0.000	0.0 ± 0.000	1134.0 ± 0.000	0.5± 0.000

Figure S11. Absorbance rate of change and wavelength peak shift of dsDNA- HiPco (9,4) SWNT Complex by H_2O_2

When the H_2O_2 concentration changed from 49 μM to 9.8 mM in step by step, the rate of change in absorbance increased, but at 98 mM, the rate of change decreased. The rate of change at 0.98mM was -7.20%. %. In the same range, recovery of absorbance by addition of catechin was observed. The rate of change at 0.98mM was 7.88%.

No significant change was observed in the wavelength peak shift.

The data are presented as the average of three independent experiments.

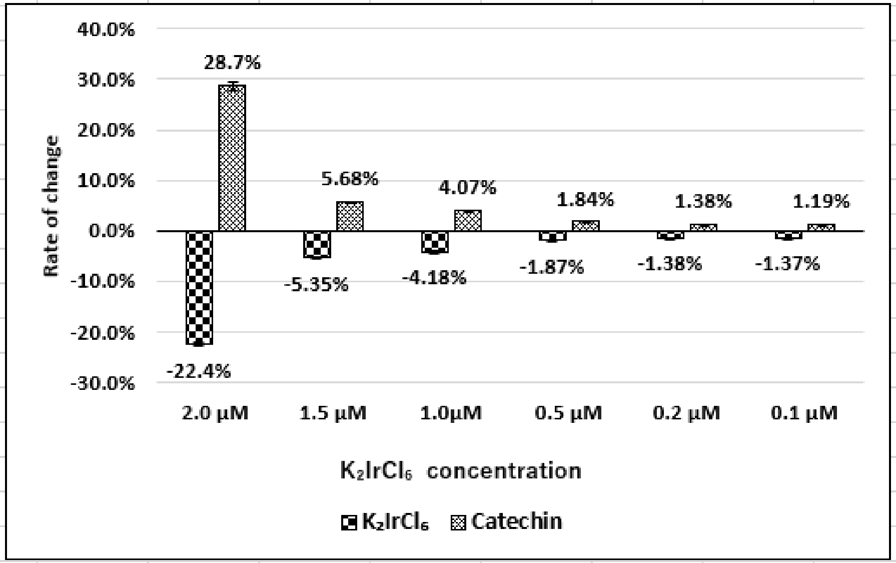

Concentration	dsDNA- HiPco (9,4) SWNT Absorbance				
(K_2IrCl_6)	Initial State	K_2IrCl_6 Addition	Change rate from Initial state	Catechin addition	Change rate from H_2O_2 Addition
2.0 µM	0.5430 ± 0.0002	0.4212 ± 0.0034	-22.4 ± 0.49%	0.5422 ± 0.0000	28.7± 0.84%
1.5 µM	0.5417 ± 0.0002	0.5128 ± 0.0012	-5.35 ± 0.15%	0.5419 ± 0.0000	5.68 ± 0.20%
1.0 µM	0.5404 ± 0.0033	0.5178 ± 0.0006	-4.18 ± 0.40%	0.5389 ± 0.0001	4.07 ± 0.08%
0.5 µM	0.5404 ± 0.0003	0.5303 ± 0.0002	-1.87 ± 0.02%	0.5401 ± 0.0000	1.84 ± 0.02%
0.2 µM	0.5389 ± 0.0002	0.5315 ± 0.0001	-1.38 ± 0.01%	0.5388 ± 0.0000	1.38 ± 0.01%
0.1 µM	0.5388 ± 0.0005	0.5314 ± 0.0001	-1.37 ± 0.07%	0.5377 ± 0.0000	1.19 ± 0.00%

Concentration	dsDNA- HiPco (9,4) SWNT Peak Wavelength (nm)				
(K_2IrCl_6)	Initial State	K_2IrCl_6 Addition	Shift from Initial state	Catechin addition	Shift from Initial state
2.0 µM	1134.5 ± 0.000	1132.0 ± 0.000	-2.5 ± 0.000	1135.2 ± 0.236	0.7± 0.192
1.5 µM	1134.5 ± 0.000	1134.5 ± 0.000	0.0 ± 0.000	1135.5 ± 0.000	1.0± 0.000
1.0 µM	1134.5 ± 0.000	1134.5 ± 0.000	0.0 ± 0.000	1135.0 ± 0.000	0.5 ± 0.000
0.5 µM	1134.5 ± 0.000	1134.5 ± 0.000	0.0 ± 0.000	1135.0 ± 0.000	0.5 ± 0.000
0.2 µM	1134.5 ± 0.000	1134.5 ± 0.000	0.0 ± 0.000	1135.0 ± 0.000	0.5 ± 0.000
0.1 µM	1134.5 ± 0.000	1135.0 ± 0.000	0.5 ± 0.000	1135.5 ± 0.000	1.0 ± 0.000

Figure S12. Absorbance rate of change and wavelength peak shift of dsDNA- HiPco (9,4) SWNT Complex by $K_2I_rCl_6$

The decrease in absorbance proportional to the concentration was detected in the concentration range of 0.1 to 2.0 µM. The rate of change at 2.0µM was -22.4%. In the same range, recovery of absorbance by addition of catechin was observed. The rate of change at 2.0µM was 28.7%. At the concentration of $K_2I_rCl_6$ of 2.0µM, the peak wavelength was shifted 2.5 nm to the short wavelength side. By adding catechin, it recovered to near the initial state with the addition of catechin.

The data are presented as the average of three independent experiments.

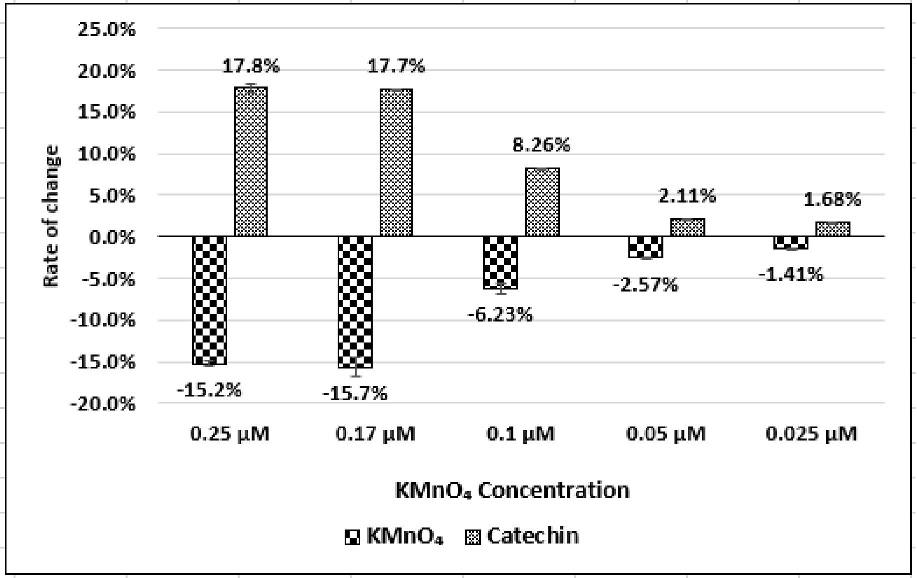

Concentration	dsDNA- HiPco (9,4) SWNT Absorbance				
($KMnO_4$)	Initial State	$KMnO_4$ Addition	Change rate from Initial state	Catechin addition	Change rate from H_2O_2 Addition
0.25 µM	0.3845 ± 0.0001	0.3260 ± 0.0016	-15.2 ± 0.33%	0.3841 ± 0.0000	17.8 ± 0.47%
0.17 µM	0.3889 ± 0.0069	0.3280 ± 0.0002	-15.7 ± 1.20%	0.3861 ± 0.0000	17.7 ± 0.03%
0.10 µM	0.3814± 0.0037	0.3576 ± 0.0003	-6,23 ± 0.01%	0.3871 ± 0.0000	8.26 ± 0.06%
0.05 µM	0.4041 ± 0.0000	0.3937 ± 0.0001	-2.57 ± 0.01%	0.4021 ± 0.0000	2.21 ± 0.01%
0.025 µM	0.3947 ± 0.0001	0.3981 ± 0.0000	-1.41 ± 0.00%	0.3957 ± 0.0000	1.68 ± 0.00%

Concentration	dsDNA- HiPco (9,4) SWNT Peak Wavelength (nm)				
($KMnO_4$)	Initial State	$KMnO_4$ Addition	Shift from Initial state	Catechin addition	Shift from Initial state
0.25 µM	1134.7 ± 0.236	1133.2 ± 0.236	-1.5 ± 0.000	1135.3 ± 0.000	0.7 ± 0.000
0.17 µM	1134.0 ± 0.000	1133.5 ± 0.000	-0.5 ± 0.000	1134.5 ± 0.000	0.5 ± 0.000
0.10 µM	1134.0 ± 0.000	1134.5 ± 0.000	0.5 ± 0.000	1134.5 ± 0.000	0.5 ± 0.000
0.05 µM	1134.0 ± 0.000	1134.5 ± 0.000	0.5 ± 0.000	1134.5 ± 0.000	0.5 ± 0.000
0.025 µM	1134.0 ± 0.000	1134.5 ± 0.000	0.5 ± 0.000	1135.0 ± 0.000	1.0 ± 0.000

Figure S13. Absorbance rate of change and wavelength peak shift of dsDNA- HiPco (9,4) SWNT Complex by KM_nO_4

The decrease in absorbance proportional to the concentration was detected in the concentration range of 0.025 to 0.1 µM. The rate of change at 0.25µM was -15.2%. In the same range, recovery of absorbance by addition of catechin was observed. The rate of change at 0.25µM was 17.8%. At the concentration of KM_nO_4 of 0.25µM, the peak wavelength was shifted 1.5 nm to the short wavelength side. By adding catechin, it recovered to near the initial state with the addition of catechin.

The data are presented as the average of three independent experiments.

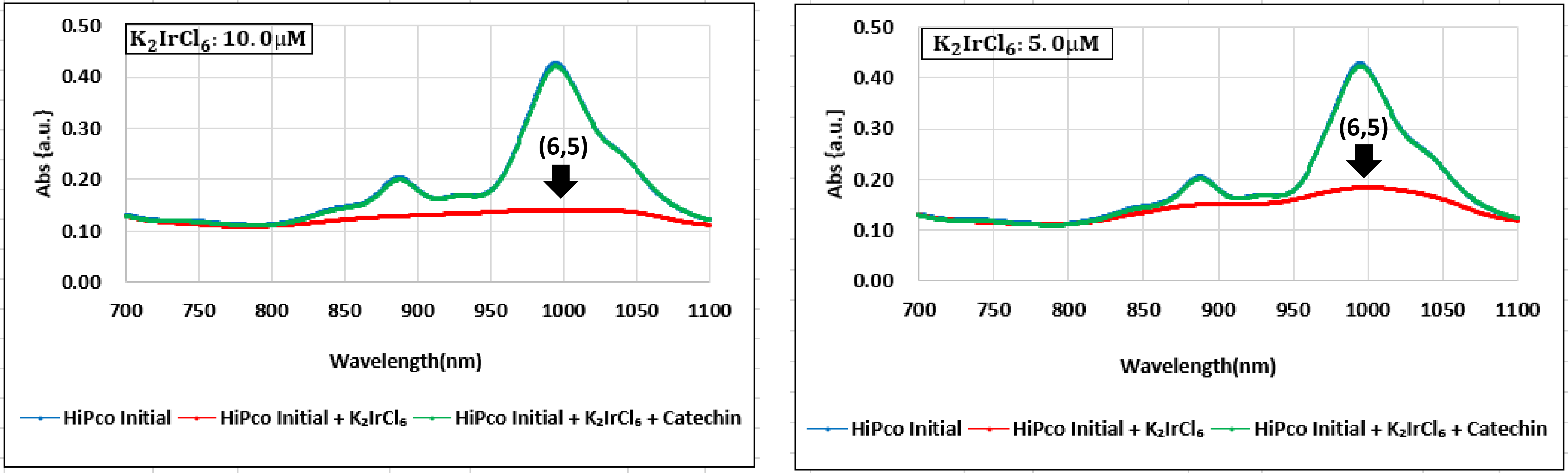

(a) (b)

Figure S14. Absorbance spectra of dsDNA- dsDNA- enriched (6,5) SWNT Complex by $K_2I_rCl_6$

(a) Absorption spectra of the dsDNA- enriched (6,5) SWNT complex following the addition of $K_2I_rCl_6$(10.0μM) and catechin.

(b) Absorption spectra of the dsDNA- enriched (6,5) SWNT complex following the addition of $K_2I_rCl_6$(5.0μM) and catechin.

The spectral peak was slightly detected at 5.0 μM, but no spectral peak was observed at 10.0 μM.

The data are presented as the average of three independent experiments.

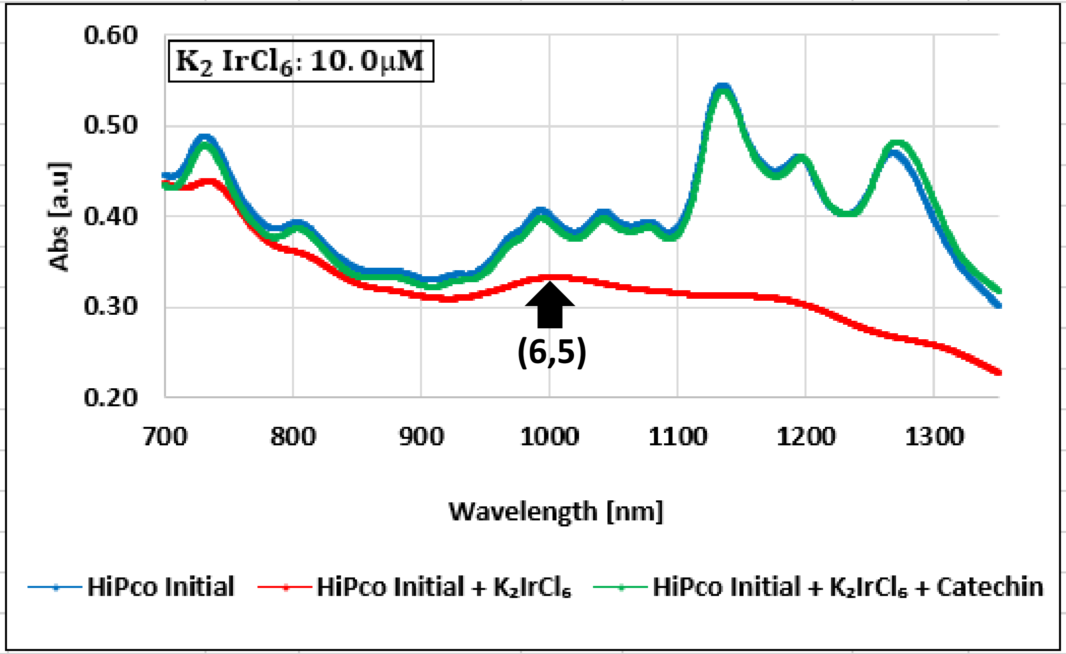

(a)

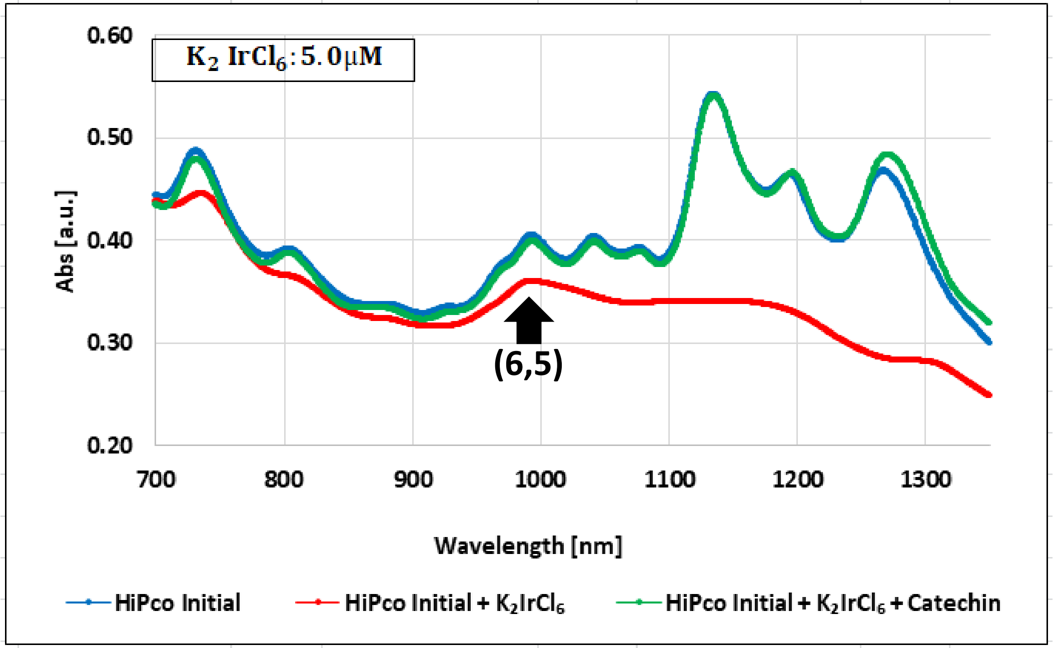

(b)

Figure S15. Absorbance spectra of dsDNA- HiPco (6,5) SWNT Complex by $K_2I_rCl_6$

(a) Absorption spectra of the dsDNA- HiPco (6,5) SWNT complex following the addition of $K_2I_rCl_6$(10.0µM) and catechin.
(b) Absorption spectra of the dsDNA- HiPco (6,5) SWNT complex following the addition of $K_2I_rCl_6$(5.0µM) and catechin.
The spectral peak was slightly detected at the concentration of 5.0 µM, but no spectral peak was observed at 10.0 µM.
The data are presented as the average of three independent experiments.

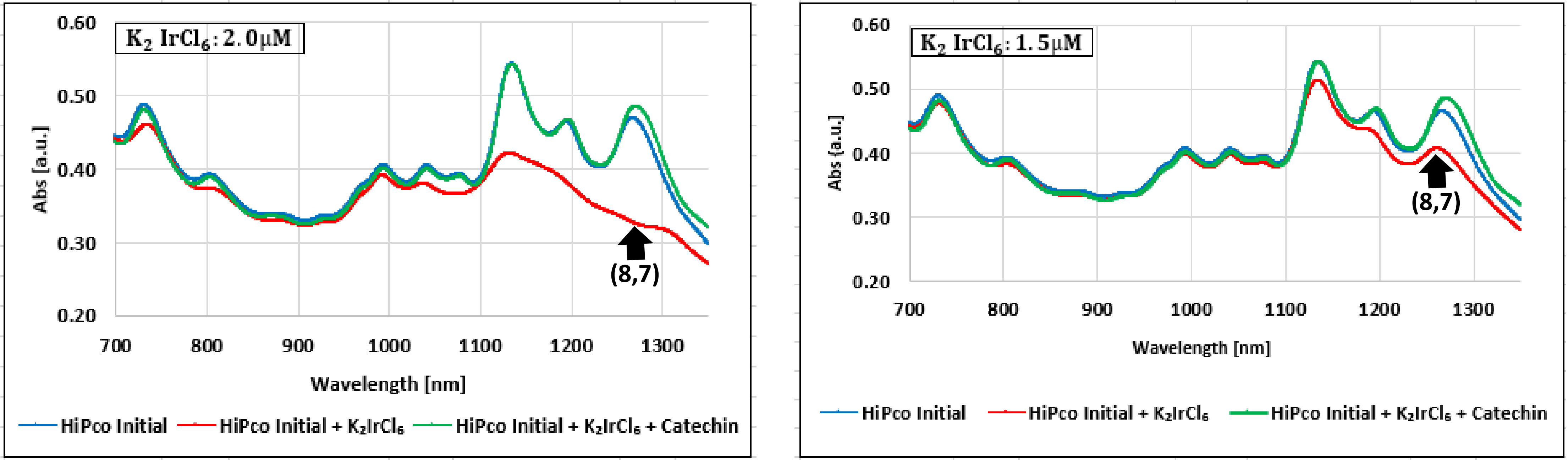

(a) (b)

Figure S16. Absorbance spectra of dsDNA- HiPco (8,7) SWNT Complex by $K_2I_rCl_6$

(a) Absorption spectra of the dsDNA- HiPco (8,7) SWNT complex following the addition of $K_2I_rCl_6$(2.0μM) and catechin.

(b) Absorption spectra of the dsDNA- HiPco (8,7) SWNT complex following the addition of $K_2I_rCl_6$(1.5μM) and catechin.

The spectral peak was detected at the concentration of 1.5 μM, but no spectral peak was observed at 2.0 μM.

The data are presented as the average of three independent experiments.

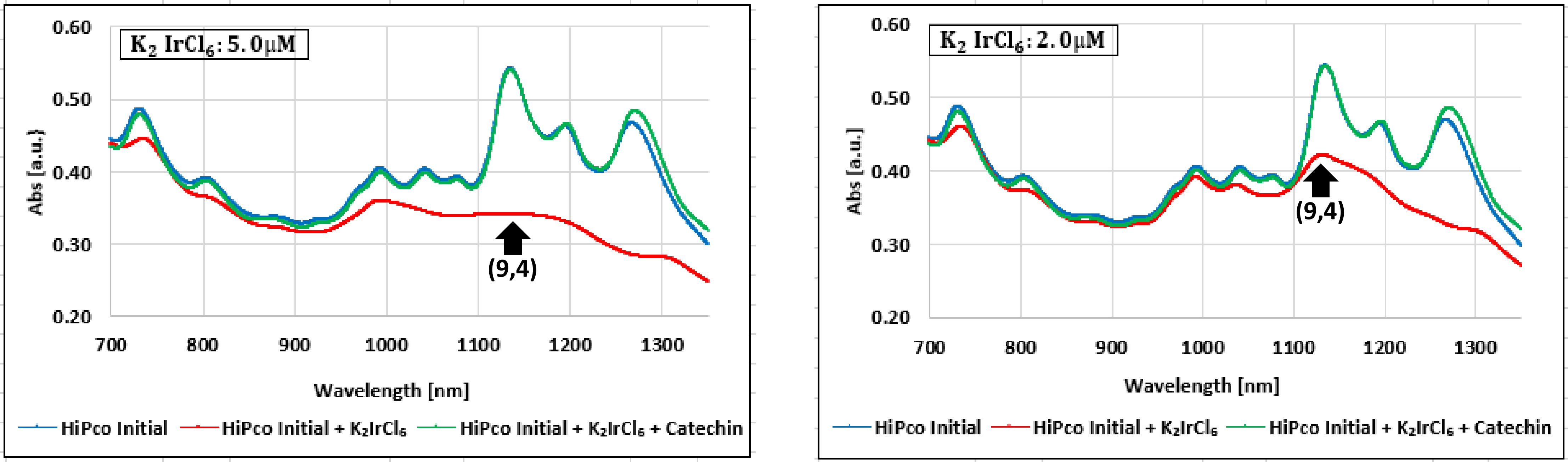

(a)

(b)

Figure S17. Absorbance spectra of dsDNA- HiPco (9,4) SWNT Complex by $K_2I_rCl_6$

(a) Absorption spectra of the dsDNA- HiPco (9,4) SWNT complex following the addition of $K_2I_rCl_6$(2.0μM) and catechin.
(b) Absorption spectra of the dsDNA- HiPco (9,4) SWNT complex following the addition of $K_2I_rCl_6$(5.0μM) and catechin.
The spectral peak was detected at the concentration of 2.0 μM, but no spectral peak was observed at 5.0 μM.
The data are presented as the average of three independent experiments.

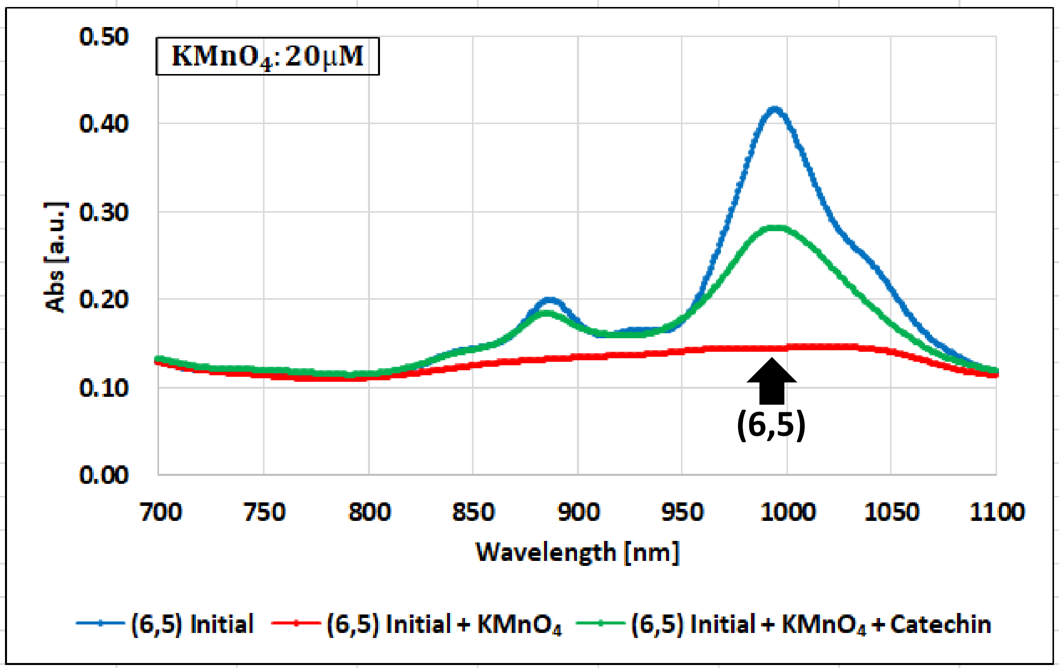

(a)

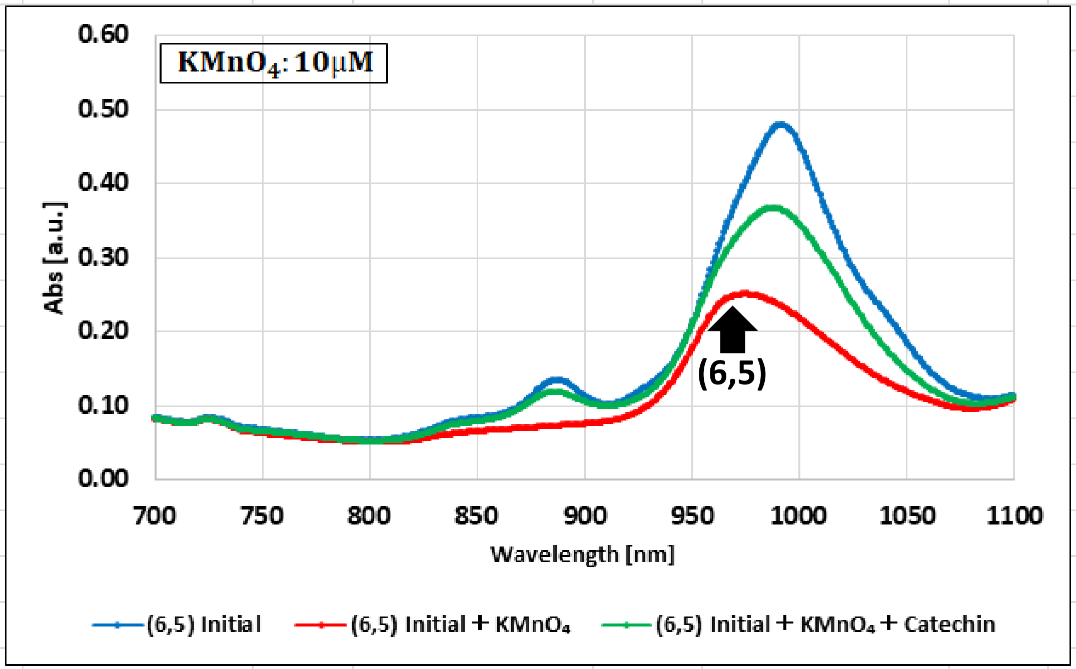

(b)

Figure S18. Absorbance spectra of dsDNA- (6,5) enriched SWNT Complex by KM_nO_4

(a) Absorption spectra of the dsDNA- (6,5) enriched SWNT complex following the addition of KM_nO_4(20μM) and catechin.
(b) Absorption spectra of the dsDNA- (6,5) enriched SWNT complex following the addition of KM_nO_4(10μM) and catechin.
The spectral peak was detected at the concentration of 10 μM, but no spectral peak was observed at 20 μM.
The data are presented as the average of three independent experiments.

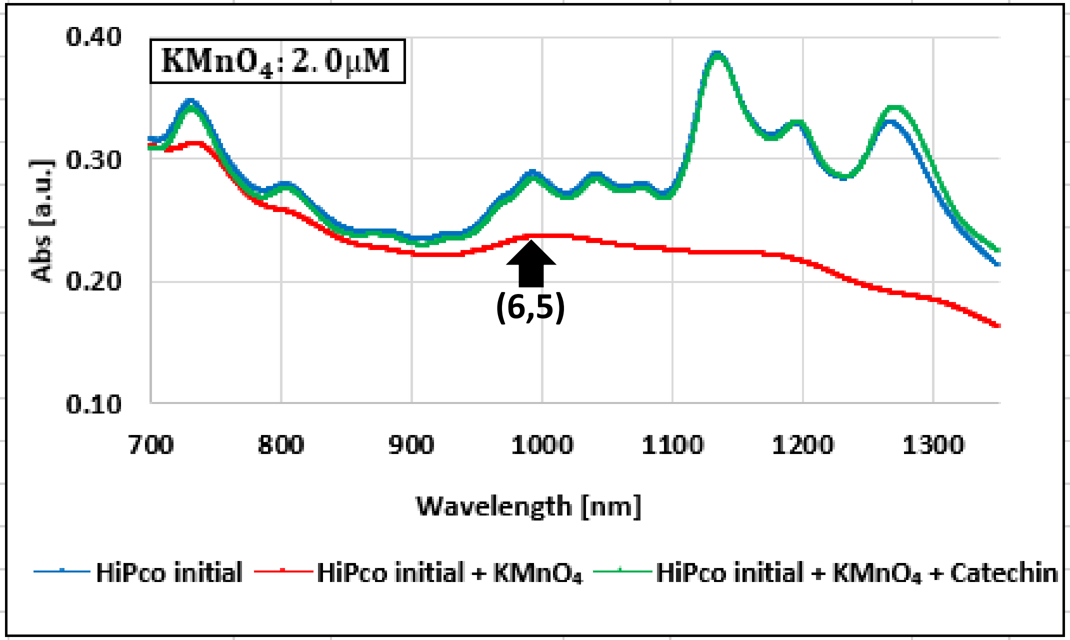

(a)

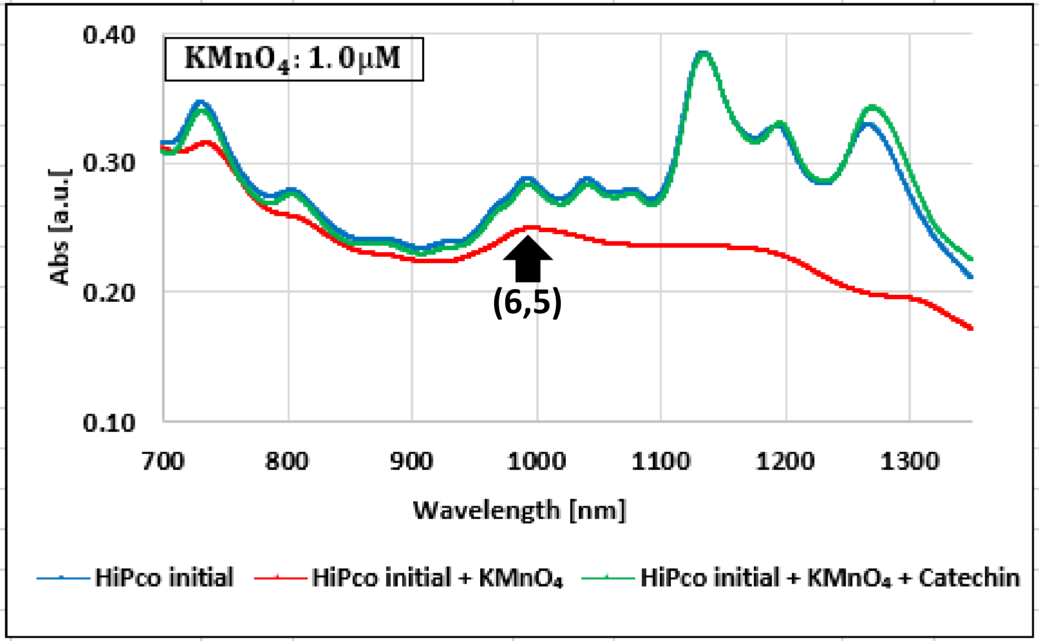

(b)

Figure S19. Absorbance spectra of dsDNA-HiPco (6,5) SWNT Complex by KM_nO_4

(a) Absorption spectra of the dsDNA- HiPco (6,5) SWNT complex following the addition of KM_nO_4(2.0μM) and catechin.

(b) Absorption spectra of the dsDNA- HiPco (6,5) SWNT complex following the addition of KM_nO_4(1.0μM) and catechin.

The spectral peak was detected at the concentration of 1.0 μM, but no spectral peak was observed at 2.0 μM.

The data are presented as the average of three independent experiments.

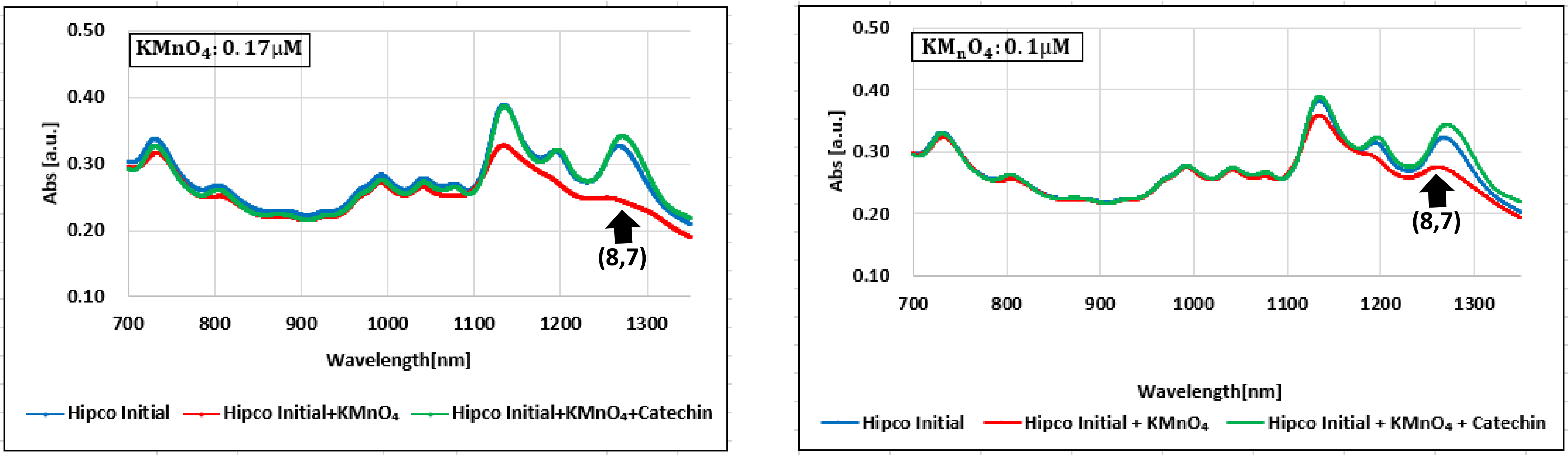

(a) (b)

Figure S20. Absorbance spectra of dsDNA-HiPco (8,7) SWNT Complex by KM_nO_4

(a) Absorption spectra of the dsDNA- HiPco (8,7) SWNT complex following the addition of KM_nO_4(0.17μM) and catechin.

(b) Absorption spectra of the dsDNA- HiPco (9,4) SWNT complex following the addition of KM_nO_4(0.1μM) and catechin.

The spectral peak was detected at the concentration of 0.1 μM, but no spectral peak was observed at 0.17 μM.

The data are presented as the average of three independent experiments.

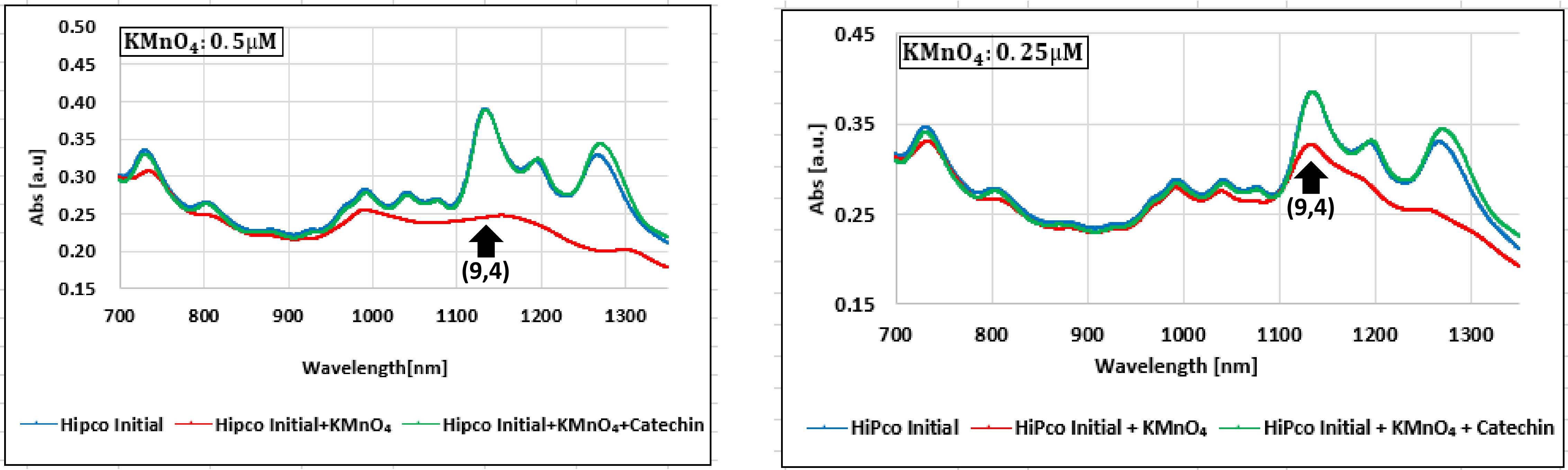

(a) (b)

Figure S21. Absorbance spectra of dsDNA-HiPco (9,4) SWNT Complex by KM_nO_4

(a) Absorption spectra of the dsDNA- HiPco (9,4) SWNT complex following the addition of KM_nO_4(0.50μM) and catechin.

(b) Absorption spectra of the dsDNA- HiPco (9,4) SWNT complex following the addition of KM_nO_4(0.25μM) and catechin.

The spectral peak was detected at the concentration of 0.25 μM, but no spectral peak was observed at 0.50 μM.

The data are presented as the average of three independent experiments.
